# Exploring the involvement of serine proteases in neutrophil extracellular traps: a review of mechanisms and implications

**DOI:** 10.1038/s41419-025-07857-w

**Published:** 2025-07-18

**Authors:** Aleksandra Korba-Mikołajczyk, Katarzyna Dominika Służalska, Paulina Kasperkiewicz

**Affiliations:** Department of Chemical Biology and Bioimaging, Wroclaw University of Science and Technology, Wroclaw, Poland

**Keywords:** Cell death, Proteolysis

## Abstract

Neutrophils play a critical role in the first-line of defense against circulating pathogens and contain a wide array of granules that store antimicrobial proteins, with neutrophil serine proteases (NSPs) and defensins serving as crucial components. NSPs such as neutrophil elastase (NE), proteinase 3 (PR3), cathepsin G (CatG) and neutrophil serine protease 4 (NSP4) exhibit distinct substrate specificities that underpin their critical roles in immune defense and inflammation [1]. After neutrophils are activated, they form and release neutrophil extracellular traps (NETs) consisting of decondensed chromatin and intracellular proteins through a process called NETosis, which leads to neutrophil death. Although NETosis is predominantly categorized as a suicidal process, several studies have suggested that neutrophils remain viable after NETosis under certain circumstances. To date, research has focused on the mechanisms underlying NETosis and roles of various factors such as reactive oxygen species (ROS), nicotinamide adenine dinucleotide phosphate (NADPH), myeloperoxidase (MPO), and peptidyl arginine deiminase 4 (PAD4). Metabolic pathways such as glycolysis are critical for NET formation, with exogenous glucose and glutamine enhancing NET release. Neutrophils cultured in glucose-free conditions fail to undergo NETosis upon phorbol-12-myristate-13-acetate (PMA) stimulation. ROS-mediated signaling promotes NE release from the azurosome, F-actin degradation, and NE translocation to the nucleus, facilitating chromatin decondensation. Notably, rapid F-actin disassembly has similarly been observed during NETosis induced by PMA and ionomycin. Recently, the role of NSPs during NET formation and their extracellular functions have received increased attention from researchers. The exact mechanism of NET formation remains unknown, and the process itself still raises controversies regarding its overlapping aspects with other forms of cell death, the role of NSPs, the nature of scaffolding DNA, and the possible involvement of other factors. Here, we discuss the intricate pathways governing NET formation, outline the diverse enzymes and proteins crucial for NET assembly, and highlight potential mechanisms controlling NET release. We pay particular attention to the regulation of NSP proteolytic activity and the nuanced role of NSPs during processes such as degranulation, which can be classified as extracellular mechanisms associated with NET formation. Dysregulated NETosis and NSP activity have been implicated in pathological states and diseases. Therefore, understanding the functions of NSPs and their role in NET formation might facilitate the development of new diagnostic and therapeutic strategies.

## Facts

- Neutrophil extracellular trap (NET) formation is a process through which extracellular fibers composed of DNA and a large variety of both cytosolic and nuclear proteins and peptides are released by neutrophils and other types of immune cells, leading to neutrophil death.
- NADPH participates in NET formation which in turn plays a role in trapping, immobilizing, and killing a broad spectrum of pathogens.
- Dysregulated NET formation leads to pathological stages and diseases.

## Remaining questions

- What are the distinguishing characteristics of different forms of NETotic cell death?
- Are neutrophil serine proteases (NSPs) encapsulated within neutrophil extracellular traps (NETs) functionally active?
- By what mechanisms is the activity of neutrophil serine proteases (NSPs) in NETs regulated?
- Do NSPs participate in NET formation?
- Can the modulation of neutrophil serine protease (NSP) activity serve as an effective therapeutic strategy?

## Elucidating NETosis: neutrophil extracellular traps (NETs) in action

When activated during infection, neutrophils undergo a remarkable process known as NETosis, wherein they release extracellular fibers composed of DNA and proteins. In this text, we use the term “NETosis” exclusively to refer to programmed cell death associated with NETs extrusion, and the terms will be used interchangeably. This distinctive process, initially characterized as a novel form of cell death, stands in stark contrast to apoptosis, necroptosis, and pyroptosis [2]. Moreover, in addition to their nucleic acid content, NETs can contain more than 20 known proteins, including histones, neutrophil serine proteases (NSPs), myeloperoxidase (MPO), actin, defensins, and protein kinase C (PKC) [3].

Neutrophils, as well as other granulocytes such as mast cells, release extracellular traps (ETs) [4]. NETs are consistently released in response to diverse stimuli, such as antibodies and immune complexes, chemokines, cytokines such as TNF-alpha and IL-8, and microcrystals [5].

NETosis can be induced by various stimuli, including potent inducers such as PMA, living gram-negative and gram-positive bacteria, and lipopolysaccharide (LPS) and less potent inducers such as activated platelets and fungi, providing a spectrum of conditions under which NETosis can occur [6]. While the literature presents varying results on NETosis induced by different stimuli, PMA and calcium ionophores consistently exhibit high success rates [2, 6].

Intriguingly, despite nearly two decades of study, the primary function of NETs remains incompletely understood. The evidence points toward their likely role in trapping, immobilizing, and eliminating a spectrum of pathogens, including gram-positive and gram-negative bacteria, acid-resistant bacteria, fungi [7], protozoa, and influenza viruses [2, 8, 9].

Additionally, recent work by our group has indicated that NSPs released during degranulation following NET formation are inactive. A study using activity-based probes (ABPs), which label only the active form of the enzyme, combined with antibodies revealed the presence of NSPs, including NE, PR3, and CatG, in NETs; however, these proteins were found to be in their inactive confirmation. These findings suggest that NSPs in polymorphonuclear neutrophils (PMNs) have different functions, such as activating inflammatory responses rather than trapping or killing pathogens [10].

In terms of metabolic requirements and cytoskeleton, there are also things that need to be met in order for neutrophils to release NETs properly [11, 12]. Metabolic processes such as glycolysis are essential for NET formation; notably, the presence of exogenous glucose and glutamine has also been shown to promote NET release, as investigated in 2014 by Snchez-Garcia’ group [13]. To investigate the metabolic requirements for NET formation, neutrophils were cultured in RPMI-1640 media lacking glucose, glutamine, or both, with complete medium as a control. PMA stimulation for 3 h induced robust NET formation in glucose-containing media, as confirmed by fluorescence and electron microscopy. Glucose deprivation completely abrogated NET release, whereas glutamine absence partially reduced it [13]. Quantification of extracellular DNA area revealed a significant reduction in NET formation under glucose-free conditions. ROS production, assessed 1 h post-stimulation, paralleled NET formation patterns, confirming the ROS-dependence of the process. To further probe glucose metabolism, neutrophils were treated with 2-deoxyglucose (2-DOG) or oligomycin. 2-DOG nearly abolished NET formation, while oligomycin had a modest inhibitory effect, indicating that NETosis relies primarily on glycolysis rather than mitochondrial ATP production [13]. The involvement of the actin cytoskeleton in NETosis was first proposed by Metzler et al. [14], who demonstrated that ROS production following neutrophil stimulation leads to NE release from the azurosome, subsequent F-actin degradation, and NE translocation to the nucleus to facilitate chromatin decondensation. During NETosis in response to *Candida albicans*, neutrophils underwent depolarization and actin cytoskeleton disassembly, coinciding with nuclear decondensation. This abrupt shutdown of actin dynamics prompted investigation into the role of NE. Inhibition of NE activity prevented its nuclear translocation and resulted in its cytoplasmic accumulation, away from azurophilic granules. NE-inhibited neutrophils-maintained polarization and active chemotaxis, unlike control cells, and developed large filopodia with NE-actin colocalization. In vitro assays confirmed that NE binds F-actin in the presence of NE inhibitor, suggesting that NE must degrade actin to become soluble and translocate to the nucleus [14]. Consistently, actin levels in *C. albicans*-stimulated neutrophils decreased rapidly, correlating with NE translocation. These findings highlight a dual role of NE in both chromatin decondensation and actin remodeling, suggesting that NE-mediated cytoskeletal breakdown is essential for halting migration and enabling NET release at infection sites [14]. Similar rapid disassembly of F-actin has been observed during NETosis induced by PMA and ionomycin [15].

Notably, this process is commonly observed in contexts such as cancer, inflammation, and infections, especially lung infections caused by viruses [8]. In the context of cancer metastasis, NET formation may initiate cascading events that involve blood components, potentially facilitating tumor progression [16]. Moreover, NETs are promising biomarkers for certain cancer types, as indicated by Demer’s group, who proposed a correlation between elevated plasma CitH3 levels and adverse outcomes [17]. Parallel findings have suggested that the proportion of NETs and G-CSF levels in the blood are correlated with neck and head cancer progression [18]. Tumor cells further promote NET formation by recruiting neutrophils to the tumor microenvironment through the release of chemoattractants such as the chemokine G-CSF.

### The inner workings: exploring the mechanisms underlying NETosis

NETosis is considered a specialized type of cell death that occurs in neutrophils. This intricate phenomenon, first discovered in 2004 by the Zychlinsky group [2], has since become the subject of intense and ongoing research, revealing multifaceted aspects of NET biology. NETs, comprising extracellular fibers extruded from neutrophils, are rich in nuclear or mitochondrial DNA [19]. Upon activation, neutrophils undergo a series of intricate morphological changes, becoming flatter in shape. The disintegration of lobular nuclei and the disappearance of internal membranes lead to the amalgamation of cellular and nuclear components [20]. This amalgamation is subsequently extruded into the environment, forming NETs and culminating in cell death.

In 2009, Simons’ group provided early evidence suggesting that the DNA associated with live neutrophils undergoing NETosis is of mitochondrial origin, whereas nuclear DNA was detected only in association with dead neutrophils [21]. These findings, based on PCR analyses, hinted at a previously unrecognized form of NET release distinct from the canonical suicidal NETosis. Building upon this concept, the phenomenon of “vital” NETosis was formally described in 2012 by the Kubes group. Using in vivo imaging during *S. aureus* infection, they demonstrated that neutrophils could release NETs while remaining viable, retaining essential functions such as crawling and phagocytosis. This paradigm-shifting observation proposed that NETosis may, under certain conditions, represent a non-lethal process involving the release of mitochondrial DNA, thereby broadening the understanding of NET biology. The visible release of DNA upon PMA or ionomycin treatment in a classic model of NETosis typically occurs within 30 min after induction [2].

However, in 2012, the phenomenon of “vital” NETosis was first elucidated by the Kubes group [22], who visualized NETs in vivo during *S. aureus* infection. In “vital” NETosis, neutrophils that release NETs during infection remain viable and are capable of phagocytosis and crawling. This unexpected discovery suggested that NETosis might also be a vital process involving the release of mitochondrial DNA. The results of another group supported these findings. Additionally, several years earlier in 2009, Simons’ group presented PCR test results indicating that the DNA surrounding live neutrophils was mitochondrial DNA, with nuclear DNA present only around dead neutrophils [21].

The visible release of DNA upon PMA or ionomycin treatment in a classic model of NETosis typically occurs within 2–8 h [23–25]. Thus, the DNA released from the cell is visible as NETs. On the other hand, the process of “vital” NETosis involves the rapid release of DNA, usually within 5–60 min after neutrophil stimulation, and is associated with the contact of cells with TLR4-activated platelets [24]. This type of NET release occurs mainly after exposure to pathogens, such gram-negative bacteria and other pathogens with microbial-specific molecular patterns that are recognized by host pattern recognition receptors. Interestingly, during “vital” NETosis, the neutrophil membrane remains intact, and dyes such as SYTOX green [26] are unable to penetrate the cell, even after NET release [18]. Using electron microscopy, Kubes’ laboratory demonstrated that “vital” NETosis can take place without the rupture of cells through the visualization of DNA trapped in vesicles that bud from the nuclear envelope and are released outside the cell [22].

The greatest controversy around this topic is the fact that the results of the majority of studies imply that a neutrophil that has lost its DNA through rupture of the cell membrane cannot function properly; therefore, it cannot perform its basic functions, including chemotaxis, phagocytosis, and leukocyte recruitment. However, if we hypothesize that neutrophils may be divided into subgroups that undergo “vital” and suicidal NETosis, it may better reflect the actual situation. Statistically, approximately 20–25% of neutrophils undergo suicidal NETosis and release NETs [22]. Keeping in mind the functional and phenotypical heterogeneity of neutrophils [27], we hypothesize that some neutrophil populations perform NETosis to entrap pathogens, whereas other subgroups are involved in pathogen elimination through degranulation and/or phagocytosis. The misconceptions in distinguishing the various forms of cell death associated with DNA extrusion have been nicely described previously [28]. For instance, morphologically similar to NETosis processes involving the release of DNA followed by cell death are: leukocytic hypercitrullination [29, 30], mtDNA expulsion [21, 31], and oxidized-mtDNA extrusion [31, 32]. In canonical ROS-dependent NETosis exhibits decreased citrullination and mtROS generation, while other forms of DNA extrusion do or do not require ROS, citrullination, mtROS production, or the knowledge about those aspects is unknown. The available data also do not clarify whether these forms of cell death serve the same functions; however, the assumption that NETosis acts mostly anti-microbial while other types can exhibits rather autoimmunogenic or anti-inflammatory properties seems to be appropriate [28]. Considering that the concept of programmed cell death represents highly heterogenous processes, with mixed morphotypes and overlapping biochemical parameters it is extremely difficult to clearly demarcate these processes, and even more challenging to interpret data properly [33]. Because of parallels in nuclear and cytoplasmic changes NETotic cell death has been often confused with apoptosis, while analogies in disturbances of plasma membrane resulted in confusion with necrosis or pyroptosis. For instance, NETosis and pyroptosis are characterized by cell membrane disruption; however, DNA can be extruded outside the cell or retained within, respectively [10]. Additionally, it is proposed that NE-activated GSDMD facilitates nuclear pore formation allowing DNA extrusion in NETosis in contrast to caspase-1/4/5-induced GSDMS membrane pore formation during caspase-induced pyroptosis [34, 35]. Moreover, GSDME activation by caspase 3 causes mitochondria permeabilization in the process of apoptosis or necrosis [36, 37]. Further, ROS-dependent permeabilization of lysosomes and release of cathepsins in necrosis might seem like NETs formation dependent on mitochondrial ROS generation [38]. Also, pore-forming MLKL has been found to play a role in both, necrosis, and NETs [39, 40]. Recently, Zhu and co-workers have proposed epigenetic mechanism including PAD4 and GSDME that results in apoptosis that priming NET formation in neutrophils [41]. Mentioned studies highlights the complexity and convergence of deaths forms that may in turn contribute to possible misclassifications. We must remain aware of over- and/or underestimation of our results simply by the limitations of designed and conducted experiments. Sadden, despite many years of research into these issues, there is still no clear description or characteristic conclusive tests, and the proposed definitions are not always applied by the community.

**Fig. 1:**
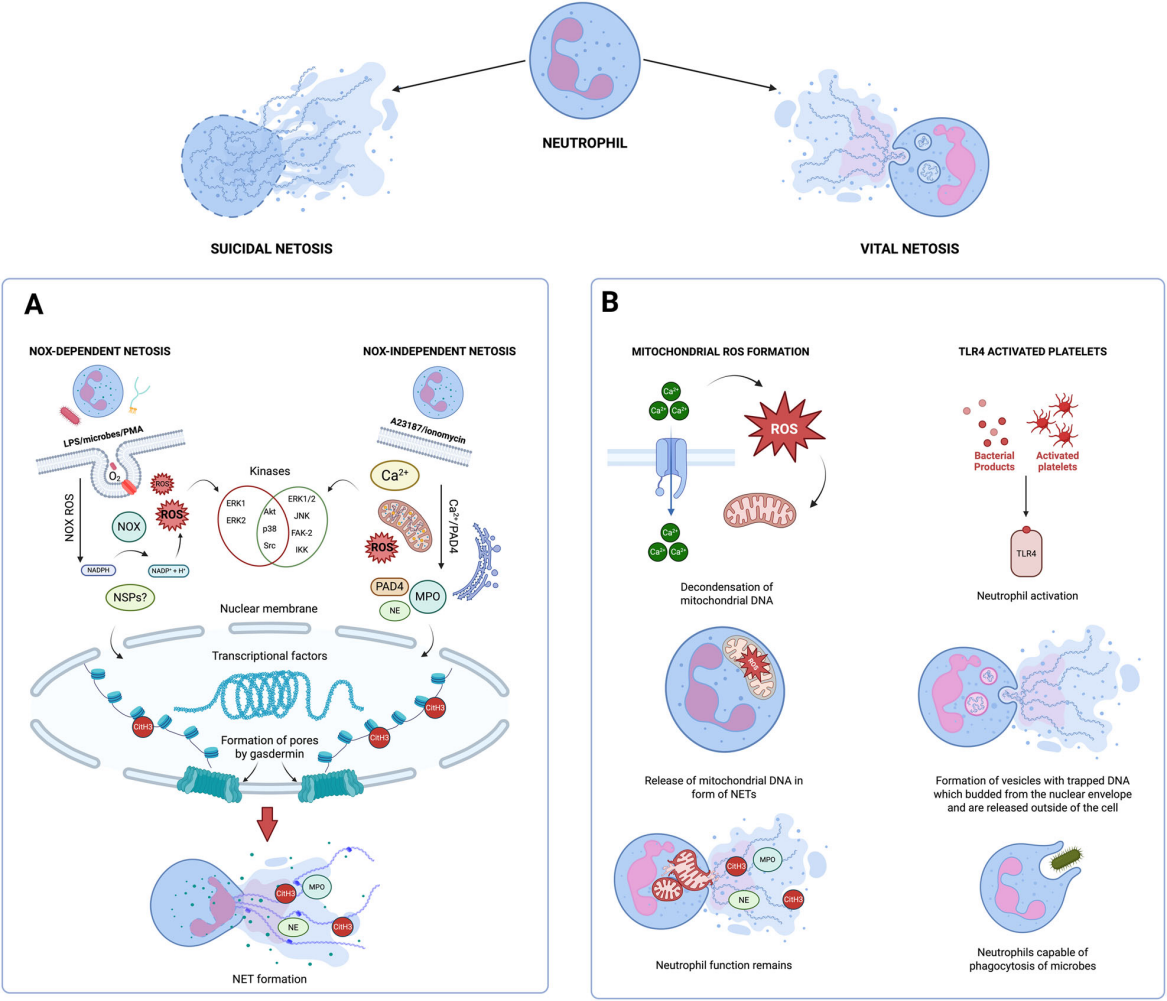
Schematic overview of NET formation, highlighting key differences between “vital” and suicidal NETosis pathways. Created with BioRender. **A** NOX-dependent NETosis is induced by PMA or LPS, while NOX-independent NETosis is triggered by calcium ionophores (A23187, ionomycin). Both pathways generate ROS and lead to chromatin decondensation and NET formation. PMA stimulation reduced full-length GSDMD, suggesting cleavage during NOX-dependent NETosis. In contrast, CGD patient neutrophils retained intact GSDMD. Occasionally, cleaved GSDMD fragments (25–30 kDa) were detected, indicating a possible link between GSDMD processing and NETosis**. B** In “vital” NETosis, often initiated by calcium influx or mitochondrial ROS, DNA is expelled via vesicular transport, allowing neutrophils to remain viable and preserve their phagocytic functions.

NETosis can be classified as “suicidal” or “vital” depending on the activation of NADPH oxidase (NOX), as shown in Fig. 1. NOX-dependent NETosis can be induced by PMA and LPS treatment. In contrast, NOX-independent NETosis is induced by the administration of calcium ionophores such as A23187 and ionomycin. Both pathways result in ROS generation via NOX, which in turn triggers kinase activation. This mobilization leads to several intermediate steps, culminating in chromatin decondensation and NET formation. In resting neutrophil nuclei, negatively charged DNA wraps around highly positively charged histones (e.g., the amino acids Arg and Lys), forming tightly compacted chromatin. Histone acetylation weakens the chromatin structure, particularly at nucleosome promoter regions, allowing protein access to DNA. In NOX-independent NETosis, increased intracellular Ca²⁺ influx activates PAD4, which translocates to the nucleus. The active form of PADI4 (PADI4: Ca²⁺ complex) deaminates arginine to citrulline in histones, leading to chromatin relaxation and transcription initiation. Neutrophils, which are activated during infection and inflammation, undergo NETosis through these complex pathways, resulting in the formation of NETs that play crucial roles in the immune response. “Vital” NETosis can be triggered by Ca^2+^ influx and the production of mitochondrial ROS, leading to the decondensation of mitochondrial DNA. This genetic material is then extruded in the form of NETs, enabling neutrophils to preserve their vital functions. Additionally, this process can be initiated by stimulation with activated platelets or bacterial components. In this form of NETosis, neutrophils generate vesicles containing DNA, which are released via membrane budding, allowing the cell to retain its phagocytic capabilities.

Recent studies also suggest the controversial connection between gasdermins and NET formation. In 2018, Zychylinsky’ group investigated the phenomenon. Activation of neutrophils with PMA has been shown to induce a reduction in full-length gasdermin D (GSDMD), suggesting proteolytic cleavage of the protein during the process of NET formation. This observation supports a potential role for GSDMD in the regulation or execution of NETosis. To further investigate the dependency of this cleavage on ROS production, neutrophils isolated from a patient with X-linked chronic granulomatous disease (CGD) were used as a control. CGD is characterized by mutations in the *CYBB* gene encoding the NOX2 subunit of NADPH oxidase, which results in defective ROS generation and an inability to undergo PMA-induced NETosis. In neutrophils derived from this CGD patient, PMA stimulation failed to reduce full-length GSDMD levels, indicating that NOX2 activity is likely required for GSDMD processing in this context [35].

These findings collectively suggest that GSDMD cleavage may be linked to NETosis in a ROS dependent manner, potentially through mechanisms that involve NOX2 activity. However, the variable presence of the cleaved product highlights the complexity of GSDMD regulation and suggests that additional factors, such as timing, cellular context, and donor-specific variability, may influence its role in neutrophil activation and NET formation. Further studies are required to elucidate the precise mechanistic contribution of GSDMD to NETosis and to determine whether its cleavage serves a functional role in membrane remodeling or chromatin release during this unique form of cell death.

Emerging evidence highlights a complex and context-dependent role for GSDMD in neutrophil cell death and NET formation. While canonical inflammasome activation via caspase-1 does not induce pyroptosis in neutrophils, stimulation of the non-canonical inflammasome through caspase-11 activation, triggered by cytosolic LPS or gram-negative bacteria, results in robust GSDMD cleavage and cell lysis. Intriguingly, caspase-11-driven neutrophil death does not mirror classical pyroptosis observed in macrophages, but instead manifests hallmark features of NETosis, including nuclear delobulation, histone citrullination, chromatin extrusion, and disruption of nuclear, granule, and plasma membranes [42].

Notably, this caspase-11–dependent form of NETosis occurs independently of neutrophil elastase (NE), myeloperoxidase (MPO), and PAD4, enzymes traditionally considered essential for NET formation, suggesting that caspase-11 and GSDMD can act as direct executors of this process. Supporting this notion, recombinant GSDMD and caspase-11 have been shown to trigger nuclear membrane rupture and chromatin relaxation in cell-free systems. Moreover, degradation of these GSDMD-driven NETs by DNase I compromises host protection in models of intracellular *Salmonella* infection, underscoring a previously unappreciated antimicrobial function of NETs generated via non-canonical inflammasome signaling [43].

In addition to caspase-mediated cleavage, GSDMD can also be processed by neutrophil elastase. While both enzymes generate functional N-terminal fragments capable of inducing lytic cell death, the biological consequences appear to vary. One study suggests that NE-mediated GSDMD cleavage promotes spontaneous cell death in aging neutrophils, whereas another proposes that this pathway facilitates NET extrusion as a mechanism of host defense. These divergent findings may reflect differences in the spatial and temporal dynamics of NE activity. High nuclear concentrations of NE during early NETosis may preferentially promote nuclear membrane disruption, while low cytosolic NE levels in aging cells may gradually activate GSDMD, leading to cell death without NET formation.

Together, these findings point to a multifaceted role for GSDMD in neutrophil biology, acting at the intersection of pyroptosis and NETosis. Given its emerging relevance in inflammatory and infectious diseases, further investigation into the regulatory mechanisms controlling GSDMD activation and function in neutrophils is warranted, particularly with respect to its potential as a therapeutic target.

Within 5–60 min of neutrophil stimulation, NETosis is initiated and can be observed by the labeling of DNA with fluorescent tags. In NOX-independent NETosis, DNA labeling is driven by an increase in the intracellular calcium concentration, which triggers PAD4-mediated histone citrullination. During this time, the DNA remains inside the nucleus and is not visible outside the cell. This contrasts with NOX-dependent NETosis, where DNA labeling typically occurs within 30–60 min. Kinases, particularly Akt, play a role in NOX-independent NETosis, whereas both Akt and MAPK pathway members, including ERK and p38, are involved in NOX-dependent NET release [44]. ROS and MPO are essential for NOX-dependent NETosis, with ROS playing dual roles in inducing DNA damage and facilitating DNA repair mechanisms, allowing for the full opening of chromatin for repair. DNA repair proteins, including poly (ADP-ribose) polymerase (PARP), proliferating cell nuclear antigen (PCNA), 9-oxoguanine DNA glycosylase (OGG1), and DNA polymerase β, are produced by neutrophils in response to NETosis [45]. NSPs are important neutrophil enzymes that play executive roles; however, their role in NETosis remains controversial. Therefore, it will be discussed in detail in the next paragraphs.

## Guardians of immunity: exploring NSPs

NSPs are key effectors in neutrophils and are stored in an active state within granules for rapid responses. As hydrolases of the protease subclass, NSPs hydrolyze amide bonds, breaking down proteins or peptides into smaller peptides or amino acids. The NSP group comprises elastase-like enzymes NE and PR3; the chymotrypsin-like enzyme CatG; and the recently identified trypsin-like enzymes neutrophil serine proteinase 4 (NSP4), Granzyme A (GrA, GZMA), and Granzyme B (GrB, GZMB). Characterized by a conserved serine (Ser) residue in the catalytic center that forms a catalytic triad together with Asp and His, NSPs undergo maturation by posttranslational modifications at both the N-terminal and C-terminal ends to attain their active form.

Neutrophils undergo a sequential granule formation process during their early development in the bone marrow, resulting in the presence of distinct granule subsets. These include mainly azurophil granules (primary granules), specific granules (secondary granules), gelatinase granules (tertiary granules), and secretory granules [46]. The allocation of proteins into these subsets is solely based on the stage in which the granules are formed and does not rely on individual sorting signals within the proteins [47]. Consequently, mature serine proteases are exclusively found in azurophil granules (all NSPs) or secondary granules (PR3), which form during the promyelocytic stage but are absent in granules formed at later stages of neutrophil development [48–50]. The mechanism by which proteases are directed into granules rather than constitutively secreted remains unclear, as no sorting motifs have been identified. Mannose-6-phosphate receptors, which are typically involved in protein sorting into granules, are not involved in neutrophil protein sorting. Instead, adapter protein 3 (AP3) is crucial for directing serine proteases to azurophil granules [31]. AP3 acts as a cargo protein, transporting proteins from the trans-Golgi network to the granule compartment. AP3 dysfunction, as observed in Hermansky–Pudlak syndrome and canine cyclic neutropenia, disrupts normal NE trafficking [32]. Recent research has indicated that serglycin, a major intracellular proteoglycan, is also essential for NE localization to granules [33, 34]. However, the mechanism by which CatG and PR3 target azurophil granules remains unidentified. Despite challenges in characterizing the sorting mechanism of NSPs into granules due to the dynamic mRNA expression patterns of NSPs during neutrophil maturation, these insights increase our understanding of NSP biology. Studies indicate that NE mutations can lead to aberrant NE localization, contributing to neutropenia [35], reduced NET formation, and diminished NE activity, impacting neutrophil function and contributing to immunodeficiency [36].

After the enzymes are formed, they remain inactive and require removal of the signaling dipeptide for activation prior to sorting into the granules. These changes are also dependent on pH. However, DPPI-mediated removal of the signal dipeptide, yielding the N-terminal pro-sequence, is crucial for NSP activation. DPPI is a cysteine exo-cathepsin, a lysosomal protease that belongs to the family of C1 papain-like cysteine peptidases [51]. Cysteine cathepsins are proteolytically inactive zymogens that contain a removable propeptide domain at their N terminal. The 60 kDa zymogen undergoes proteolysis at several sites to release the propeptide. As a result of proteolysis, a mature DPPI monomer is produced, which is composed of three tightly linked subunits. Afterward, DPPI further refines the resulting proenzymes by cleaving the N-terminal dipeptide within the endoplasmic reticulum (ER) and Golgi apparatus before packaging it into granules [52]. Additionally, DPPI activity is crucial for preventing premature NSP activation. Moreover, the processing of the carboxyl terminus is vital for proper translocation of serine proteases into primary (azurophilic) neutrophil granules [53, 54]. DPPI can be found mainly in the spleen, lungs, kidneys, liver, and hematopoietic cells. It is most active at a slightly acidic pH [55] and can be activated by Cl^−^ ions in a pH-dependent manner. H_2_N-Gly-Phe-AMC and H_2_N-Gly-Arg-AMC are the chemical substrates most commonly used to measure DPPI activity in biological samples. Mast cells and T lymphocytes, which are chemically activated with calcium ionophores in vivo, secrete DPPI into the extracellular milieu. This may suggest that DPPI is released from granule-associated proteins in which it is stored intracellularly [56]. The best characterized function of this enzyme is the processing of NSPs, including NE, PR3, CatG, GrA, and GrB, in the cells of the immune system [54]. In humans, the loss of DPPI function almost completely blocks the activity of NSPs and increases the level of zymogens in neutrophils, as demonstrated in Papillon–Lefèvre syndrome.

In conclusion, NSPs are integral to the immune response and are housed within neutrophil granules in a ready-for-action manner. The distinct granule subsets (mainly azurophil, specific, gelatinase, and secretory) facilitate the timely deployment of NSPs, underscoring their importance in proteolytic activities [57, 58]. Despite extensive research, the precise mechanisms of NSP sorting and granule targeting remain elusive, with notable gaps in understanding the roles of AP3 and serglycin. The activation of NSPs, which is mediated by DPPI, is crucial for their functional maturation, whereas mutations and NSP-related pathway dysfunction contribute to various immunodeficiencies. These insights into NSP biology not only highlight their vital role in immune defense but also pave the way for potential therapeutic interventions in related disorders.

### Precise targeting: substrate specificity of NSPs and their physiological substrates

NSPs exhibit distinct substrate specificities, which are crucial for their roles in immune defense and inflammation. Their selective proteolytic activity, which is determined by the unique structural configurations of subsites surrounding their active sites, enables the precise targeting of peptide bonds via a classic serine protease mechanism involving interactions with the hydroxyl group of the catalytic serine residue [3, 59, 60]. Proteolysis is selective on the basis of the amino acid residues adjacent to the scissile bond, termed Pn, P1, P1’, Pn’, etc., according to the Schechter and Berger nomenclature [61]. This specificity allows NSPs to degrade microbial proteins, modulate cytokines, and remodel extracellular matrix (ECM) components, contributing to infection clearance and the regulation of inflammatory responses. Dysregulation of NSP activity can lead to pathological conditions, making NSPs important therapeutic targets.

In 2013, O’Donoghue et al. presented results from positional scanning substrate combinatorial library (PS-SCL) and multiplex substance profiling by mass spectrometry (MSP-MS) analyses, in which NE showed a distinct preference for valine (Val), alanine (Ala), threonine (Thr), and isoleucine (Ile) at the P1 site. Additionally, proline (Pro) was favored at the P2 position; glutamine (Gln), glutamic acid (Glu), and methionine (Met) were favored at the P3 position; and norleucine (Nle) was favored at the P4 position [3]. The MSP-MS assay revealed that NE preferred Ile, Val, and Thr at the P1 site but showed low tolerance for asparagine (Asn), lysine (Lys), and most other amino acids with charged side chains. Furthermore, arginine (Arg), Gln, Nle, and tryptophan (Trp) were significantly enriched (*p* ≤ 0.05) at the P4, P3, P1’, and P2’ positions, respectively. These findings confirm previous studies highlighting the catalytic preferences of NE, particularly its affinity for small aliphatic amino acids at the P1 position and Pro or its derivatives in the S2 pocket [62]. This selectivity is attributed to the structural restrictions of the enzyme, such as the two Val residues limiting access to the S1 pocket. NE is a potent and abundant enzyme that cleaves various connective tissue substrates, facilitating neutrophil migration to infection sites. Patients with low circulating levels of α1-antitrypsin, which neutralizes excess NE activity, often develop severe lung emphysema due to the proteolytic degradation of lung connective tissue, particularly elastin, a natural NE substrate composed of repeating Pro-Val motifs that match those of the preferred S2-S1 pockets of NE [63]. NE primarily plays a role in the digestion of bacteria, ECM components, and necrotic tissue. Its physiologically relevant substrates include coagulation factor IX, epithelial cadherin, fibronectin, fibulin-2, heparin cofactor 2, and insulin-like growth factor-binding protein 3.

Cathepsin G (CatG), a member of the immune serine peptidase family, uniquely hydrolyzes both chymotryptic and tryptic substrates, particularly those containing phenylalanine (Phe) residues at P1 [43]. Its tryptic activity contributes to the activation of proteinase-activated receptors, complement component C3, and pro-urokinase plasminogen activator [42]. According to the MEROPS database, CatG prefers leucine (Leu), histidine (His), and Phe at the P1 position [51], whereas MSP-MS profiling indicates a preference for Nle at P2 and Val at the P3 position, with Pro fitting best in the S2 pocket. Structural analyses suggest that Glu226 at the base of the primary specificity pocket facilitates the accommodation of basic side chains in tryptic substrates and inhibitors, functioning analogously to Asp189 in classical tryptic peptidases [16]. Common substrates of CatG include A-type flagellin, angiotensin-1, and C-C motif chemokine 3 [44]. The primary role of CatG in neutrophils is bactericidal, assisting in the digestion of phagocytosed bacteria. Studies on CatG-deficient mice highlight the importance of CatG in surviving fungal and bacterial infections, such as those caused by *Aspergillus fumigatus* and *Streptococcus pneumoniae* [45, 46]. CatG deficiency, especially when combined with NE deficiency, impairs microbial eradication mechanisms and reduces neutrophil responsiveness to immune complex activation.

The substrates of PR3 are similar to those of NE, which is an effect of the structural similarity of these enzymes. The majority of substrates hydrolyzed by NE are also effectively processed by PR3, making the search for unique compounds for the study of these enzymes challenging. Biochemical investigations have demonstrated that PR3 exhibits proteolytic activity toward various ECM proteins, including elastin, fibronectin, vitronectin, laminin, and collagen, by targeting a GXXPG motif within a β-fold conformation, leading to protein degradation [64–66]. Like other NSPs, PR3 can disrupt mucus clearance by damaging the bronchial epithelium and cilia [66]. Additionally, PR3 is known to modulate various cytokine functions, influencing processes such as metabolism and inflammasome assembly. According to O’Donoghue’s research, at the P1 position, PR3 showed an approximately equal preference for Ala, Val, Thr, and Ile. Beyond the P1 subsite, PR3 demonstrated selectivity for aspartic acid (Asp) and Asn at P2 and for Nle, Leu, and glycine (Gly) at P4, P3, and P2′, respectively [3]. The following natural substrates of PR3 have been identified: interleukin-32, proteinase-activated receptor 2, cyclin-dependent kinase inhibitor 1, and angiotensinogen [67–69].

Structurally, NSP4 presents a challenge to the traditional trypsin-chymotrypsin-elastase classification paradigm. Its primary sequence around the active site suggests elastase-like specificity, yet it has a trypsin-like substrate preference, deviating from the expected enzymatic specificity [70, 71]. This discrepancy was attributed to the resolution of the protease’s 3D crystal structure, revealing an occluded pocket and a noncanonical conformation of the substrate P1 Arg sidechain stabilized by a network of solvent-exposed hydrogen bonds. Consequently, NSP4 can efficiently hydrolyze substrates with Arg- and Arg-derived side chain amino acids (such as citrulline and methylarginine) but not substrates containing Lys [72]. There is much to still be discovered regarding the substrate specificity of NSP4; however, Perera et al. reported that profiling its specificity with peptide libraries from *E. coli* revealed a preference for arginine at the P1 position; it cleaved Tyr-Arg-Phe-Arg-AMC and Ala-Pro-Nva-thiobenzyl esters [71].

GrB is a protease involved in the induction of rapid target cell death by cytotoxic lymphocytes [73]. Its proteolytic activity is highly dependent on the length and sequence of the substrate, highlighting the role of GrB as a regulatory protease. According to the MEROPS database, GrB prefers Asp at the P1 position; Gly, Pro, and Ala at the P2 position; and Glu, Gly, and serine (Ser) at the P3 position [74]. An unusual preference for cleavage after aspartate residues, a specificity unique among mammalian serine proteases shared with caspases, a family of cysteine proteases activated during apoptosis, was observed. Studies have strengthened the link between GrB and caspases, showing that GrB can cleave and activate certain caspase members [75]. This ability to activate caspases is suggested to be one of the mechanisms by which GrB mediates apoptosis in vivo through the cleavage of procaspase-3 and various other apoptosis-related substrates, including Bcl-2-associated transcription factor 1, the Bcl2 antagonist of cell death (BAD), and caspases 4, 8, 9, and 10 [55]. When secreted into the extracellular environment, GrB targets a range of proteins, including von Willebrand factor; cell surface receptors; and ECM components such as fibronectin, vitronectin, laminin, and fibrillin-1. This extracellular activity allows GrB to modulate cellular activation by cleaving both excitatory and inhibitory receptors and their ligands [63]. Thus, GrB plays a multifaceted role in regulating apoptosis and cellular interactions within the ECM.

GrA belongs to the S1-A (trypsin-fold) family of serine peptidases and is characterized by a specificity pocket at the S1 site that favors basic residues such as Arg and Lys [76]. GrA exhibits maximal activity with its preferred tetrapeptides, and compared with longer sequences, GrA has the ability to cleave single amino acid substrates but is less potent [77]. The substrate selection of GrA may be influenced by its unique ability to form a disulfide-linked homodimer, facilitated by a reactive cysteine (Cys) residue at position 93, in addition to the eight cysteines forming the four disulfide bonds typically found in trypsin-fold proteases [78, 79]. This enzyme displays specific substrate preferences, favoring basic Arg and Lys at the P1 position; Pro, Ser, and Leu at P2, and Gly, Ala, and Ser at P3. Common natural substrates include actin, glial fibrillary acidic protein, linker histone H1, core histone H2B, lamin B, Ape1, HMGB2 (a SET-complex binding protein), pro-IL-1β, and the thrombin receptor [80–83]. Unlike GrB, the substrate specificity of GrA indicates that it plays a significant role in caspase-independent cell death. This independence serves as a fail-safe mechanism when viral or tumor cell components inhibit the caspase pathway. A key characteristic of GrA-mediated apoptosis is the induction of single-stranded DNA nicks through the activation of NM23-H1, a nucleoside diphosphate kinase that plays a critical role in suppressing tumor metastasis [61, 62].

Researchers have investigated not only the substrates of NSPs based on natural amino acids but also noncoded residues to gain deeper insights into their pocket shapes. A variety of chemical protease substrates and probes with enhanced activity and specificity have been developed. However, identifying substrate sequences that exhibit true selectivity for a particular protease is challenging owing to the significant overlap in substrate specificities among different proteases. Increased enzyme selectivity can sometimes be achieved via the use of nonnatural amino acids, such as those in the hybrid combinatorial substrate library (HyCoSuL). This approach was applied in 2014 to assess the specificity of NE and again in 2015 for testing the catalytic preferences of NSP4. Subsequent studies focused on the substrate specificity of GrA, GrB, PR3, and CatG, resulting in the development of specific tools for investigating active NSPs in neutrophils and other immune cells. The results of analyses using the HyCoSuL corroborated the established specificity of NSPs for natural amino acids while demonstrating their capacity to accommodate a diverse range of unnatural structures. Notably, research by Kasperkiewicz et al. revealed that NE hydrolyzes substrates containing methionine sulfoxide at the S3 pocket more effectively than regular methionine does, suggesting its role in processing post-translationally modified proteins [84].

### First-line defenders: the role of NSPs in host defense

NSPs are currently understood to perform three primary functions. First, NSPs can directly kill bacteria. For example, NE can destroy the gram-negative bacterium *E. coli* by degrading protein A, leading to membrane damage and cell death [85]. In vivo, the combined action of NE, CatG, and PR3 can kill *S. pneumoniae* via phagocytosis [60]. Second, NSPs can hydrolyze host proteins to produce antimicrobial peptides. A notable example is PR3, which cleaves hCAP-18 to generate the antimicrobial peptide LL-37 [86]. Third, NSPs can reduce bacterial virulence by inactivating factors necessary for pathogenesis. For example, NEs can degrade the mobile proteins IcsA and IpaA-C of *S. flexneri*, preventing bacteria from spreading into the cytoplasm of neutrophils [60]. Similarly, CatG can hydrolyze adhesin clumping factor A of *S. aureus*, removing its active domain.

Among NSPs, NE, CatG, and PR3 seem to play the most crucial role in neutrophil functions; however, research by Mattila et al. [87] emphasized the potential importance of GrB in bacterial infections, with higher levels observed in neutrophils with greater bacterial content. While the role of GrA in neutrophils has not been confirmed directly, studies have reported increased extracellular levels of GrA during bacterial infections, suggesting a potential extracellular role in inflammation modulation [88].

However, neutrophils may also take part in the regulation of inflammation. Upon neutrophil activation at inflammatory sites, NSPs are abundantly secreted into the extracellular environment [59]. Some of these proteases remain bound to the external surface of the plasma membrane in an active form. Both soluble and membrane-bound NSPs can proteolytically regulate the activities of various chemokines, cytokines, growth factors, and cell surface receptors. Additionally, the secreted proteases activate lymphocytes and cleave apoptotic and adhesion molecules. The proinflammatory cytokine IL-18, which belongs to the IL-1 cytokine family, is normally present in its precursor form and is thought to be processed only by caspase-1 and caspase-3, may also be hydrolyzed by PR3 [89]. In fact, PR3 may also hydrolyze other interleukins, such as pro-IL-1β. Unlike other NSPs, PR3 activates IL-1β. This is likely because pro-IL-1β has a negatively charged residue at the P4 position and a positively charged residue at the P1′ position [59]. These residues can electrostatically interact with Lys99 and Asp61 in the active site of PR3. This was shown in mice deficient in caspase-1, which are still able to produce mature IL-1β in response to a local inflammatory stimulus [59].

PR3 can also cleave TNF-α [90], a key cytokine in inflammation, as evidenced by the successful use of anti-TNF-α treatments for Crohn’s disease, rheumatoid arthritis, and psoriasis [91]. TNF-α is processed by the membrane-bound metalloproteinase TNF-α converting enzyme (TACE) [92]. However, endogenous serine protease inhibitors have been shown to suppress the secretion of TNF-α from activated macrophages. PR3, but not NE, can cleave TNF-α into its active form in vitro. This proteolysis occurs between Val77 and Arg78, differing from the TACE cleavage sites (Val79 and Asp80), but does not significantly affect the biological activity of TNF-α [93].

PR3 promotes caspase-dependent apoptosis not only when released into the extracellular space or bound to the membrane but also when it crosses the endothelial plasma membrane. Although the mechanism of PR3 internalization remains unknown, it appears to be cell type specific, as lung epithelial cells do not internalize PR3 [93]. Researchers hypothesize that PR3 internalization is receptor dependent.

When endothelial cells are exposed to PR3, it leads to cleavage and inactivation of the transcription factor NF-κB and sustained activation of JNK. Interestingly, the inhibition of caspases does not prevent the hydrolysis of p65 NF-κB, and sequence analysis revealed that the PR3 cleavage site is unique compared with known caspase cleavage sites. Additionally, PR3 processes the cell cycle inhibitor p21 at Thr80 and Gly81, a region typically susceptible to caspase-3 cleavage [69]. However, PR3 hydrolyzes p21 independently of caspases, as shown by experiments in which a broad-spectrum caspase inhibitor was used. This cleavage results in the loss of p21 function, its exclusion from the nucleus, and the activation of apoptosis [69, 93].

It was proposed that PR3-induced apoptosis is faster because it circumvents the caspase cascade and directly cleaves p21. The resulting phagocytosis of apoptotic cells helps resolve inflammation by clearing both damaged endothelial cells and PR3 from the affected area. This ability may have evolved in immune cells such as neutrophils, enabling them to disrupt intracellular caspase cascades through proteases such as PR3, thus offering an advantage against microbes that avoid typical apoptotic signals. In autoinflammatory disorders such as Wegener’s disease, which characterized by the presence of anti-PR3 autoantibodies and systemic vasculitis, PR3-induced endothelial apoptosis provides a persuasive explanation for the widespread vasculitis observed in these conditions [94].

Moreover, NE contributes to the complement cascade through cleavage of the central complement protein C3, yielding a peptide that mimics the natural C3a anaphylatoxin. Like C3a, this NE-derived peptide exhibits antimicrobial activity against *E. faecalis* and *P. aeruginosa* [95]. However, this process requires the presence of the pneumococcal capsule, although the exact mechanism is still unclear. Interestingly, the role of NE in the eradication of the closely related organism *S. aureus* seems negligible, and the contribution of CatG in this context is poorly understood [96]. Furthermore, in addition to their proteolytic functions, NSPs might possess bactericidal activity through other mechanisms, which are not yet well understood. In respiratory diseases, NE has been found to drive harmful inflammation by digesting opsonins and opsonin receptors, degrading innate immune proteins such as lactoferrin, and inhibiting macrophage phagocytosis [97]. Also, it has been reported that NE can process cytokines like IL-36Ra, influencing immune responses and potentially exacerbating autoimmune conditions [98]. Another study has connected NE and NET with the progression of sepsis. It has been shown that alpha-1 antitrypsin targeted NE to inhibit NETs thereby protecting mice from sepsis-induced inflammation and coagulation [99].

Recent insights into the last discovered NSP4 protease [71] revealed its essential role in mast cell biology, potentially playing a redundant role in neutrophil function. AhYoung’s research highlighted the importance of NSP4 in mast cell activity, facilitating the release of optimal histamine and serotonin levels crucial for a robust immune response to inflammatory factors [100]. NSP4 is expressed during early mast cell development in bone marrow precursor cells and is critical for maintaining normal levels of histamine and serotonin in the secretory granules of developing mast cells [100]. NSP4 deficiency in mice leads to reduced levels of histamine and serotonin, resulting in reduced vascular leakage in models of passive cutaneous anaphylaxis (PCA) and acute arthritis. Therefore, NSP4 ensures that developing mast cells release optimal levels of serotonin and histamine, which are necessary to mount a robust inflammatory response to stimuli.

In summary, numerous studies have elucidated the multifaceted roles of NSPs in neutrophil functions, immune defense, and NETosis, emphasizing their intricate contributions to host defense mechanisms.

### Troublesome fighters: the role of NSPs in the disease

Owing to their multifaceted effects, NSPs, in addition to their beneficial role in host defense, may also contribute to the development of many pathological conditions. Indeed, several studies have highlighted the role of NET formation and the possible toxic effects of NSPs, especially NE, during sepsis, thrombosis, and ischemic-lung injury [97, 101–105]. Additionally, NET formation as well as the activities of NE and PAD4 during respiratory viral infections such as COVID-19 have been associated with thrombosis and lung injuries [106–108].

Recent studies have suggested that NET formation might induce neuroinflammation and complement activation in Alzheimer’s disease [109–111]. Elevated levels of NETs have been identified in the plasma and serum of Alzheimer’s patients [111], and NE and CatG have been proposed to drive neuroinflammation through the possible cleavage of amyloid beta aggregates [110]. Moreover, NE was found to trigger amyloid fibril fragmentation [112].

In addition, the NET-associated inflammatory microenvironment has been reported to facilitate tumor metastasis and progression. For example, CatC promotes NET formation, which in turn leads to the processing of IL-1β by PR3, increasing thrombospondin-1 degradation, and ultimately supporting the metastatic growth of lung cancer cells [113]. In colorectal cancer cells, NE released during NETosis promotes the migration of cells through the activation of ERK signaling [114]. Additionally, MPO and NE are associated with the progression and metastasis of triple-negative breast cancer cells [115].

NSPs have also been proposed to promote autoimmune responses in diseases such as rheumatoid arthritis [116]. The activity of MPO and NE was proposed to be the source of autoantigens [116]. Furthermore, NET inhibition reportedly alleviates pathology in type 1 diabetic mice [117]. Additionally, the serum levels of MPO, NE, PR3, and PAD4 have been studied as potential biomarkers of type 1 diabetes in humans [118].

## NSPs and NETosis: participants in the phenomenon

Over the last 20 years of research, several mechanisms for the formation of NET structures have been proposed, including global transcriptional activation that unwinds inactive chromatin [119], proteolytic cleavage of histones by NSPs such as NE [7, 14, 120], and posttranslational modifications of positively charged residues in histones, such as hypercitrullination of histones by PAD4 or stimulation of the release of DNA by calcium ionophores [121, 122]. At the beginning of NETosis research in 2004, a relationship between NE and DNA released upon neutrophil stimulation was observed [2]. Many subsequent studies have highlighted the importance of NSPs, especially NE, in the formation of NETs via a variety of experimental models, such as inhibitors in in vitro studies or genetic manipulations in mouse models; however, several studies have questioned the specificity of those inhibitors. In this study, we focused only on the contribution of NSPs to NETotic cell death.

In 2011, the Zychlinsky group investigated whether neutrophils undergoing NETosis are present in the sputum of patients suffering from cystic fibrosis (CF), a genetic disorder related to the abnormal colonization of bacteria causing lung tissue damage [123]. Notably, the exclusion criterion for this study was DNase treatment, since the administration of recombinant DNase is a common form of therapy used to dissolve sputum [124]. The group revealed that NE and MPO levels were associated with DNA release and low amounts of histone H3 but not H4, suggesting that NE can be translocated from granules into the nucleus, where it might contribute to chromatin decondensation. Moreover, the use of two inhibitors, GW311616A (for NE) and SLPI (broader spectrum), revealed that the inhibition of NE blocked histone degradation. However, GW311616A is not completely specific to NE. In another study, treatment of activated (by PMA, TNF-α, and *S. aureus*) neutrophils with SLPI significantly reduced NET formation. Moreover, the observed effect was related to the nuclear translocation of serpin, which allowed the formation of a complex with NE, consequently preventing histone H4 degradation [125]. Experiments with genetically modified SLPI^-/-^ mice have shown that, after stimulation of neutrophils with PMA or *S. aureus*, significantly more DNA is released, confirming the ability of SLPI to modulate NETs [125]. It has been suggested that intracellular SerpinB1 could also prevent chromatin decondensation via the inhibition of NE, CatG, and PR3. Neutrophils derived from serpinb1^-/-^ mice exhibited more effective NET formation, although the use of DPI (a nicotinamide peroxidase (NAPDH) oxidase inhibitor) suggested that the activity of NE in NET formation is determined by the activation stimulus (PMA/PFA vs. MIP-2) [126].

Several other studies have confirmed the role of NE, PR3, CatG, and cysteine proteases in NET formation via the use of small-molecule inhibitors in *Cryptosporidium parvum*, *Leismania*, *S. aureus*, *P. aeruginosa* or PMA induction in human neutrophils [127–131]. In 2017, the Gly201Arg mutation of NE was associated with decreased NE activity and impaired NET formation [132]. In addition, data generated from NSP-deficient models indicate various and often contradictory contributions of NE and PR3 to NET formation [133–135].

The function of NSPs is related mainly to their intracellular proteolytic action within the phagosome to disarm pathogens and virulence factors [136–139]. Additionally, released NSPs associated with NET structures are commonly thought to neutralize pathogens through their enzymatic activity [2, 20, 120]. As research has progressed, the following new roles of extracellular NSPs have been proposed: (i) promoting migration by processing ECM components such as elastin, fibronectin, or collagen; (ii) processing cytokines, growth factors, and receptors and thus modulating their activity; and (iii) initiating the response of other cells after being transferred to other locations [50, 140–148].

### Building the traps: the role of NSPs in the formation of NETs

**Fig. 2:**
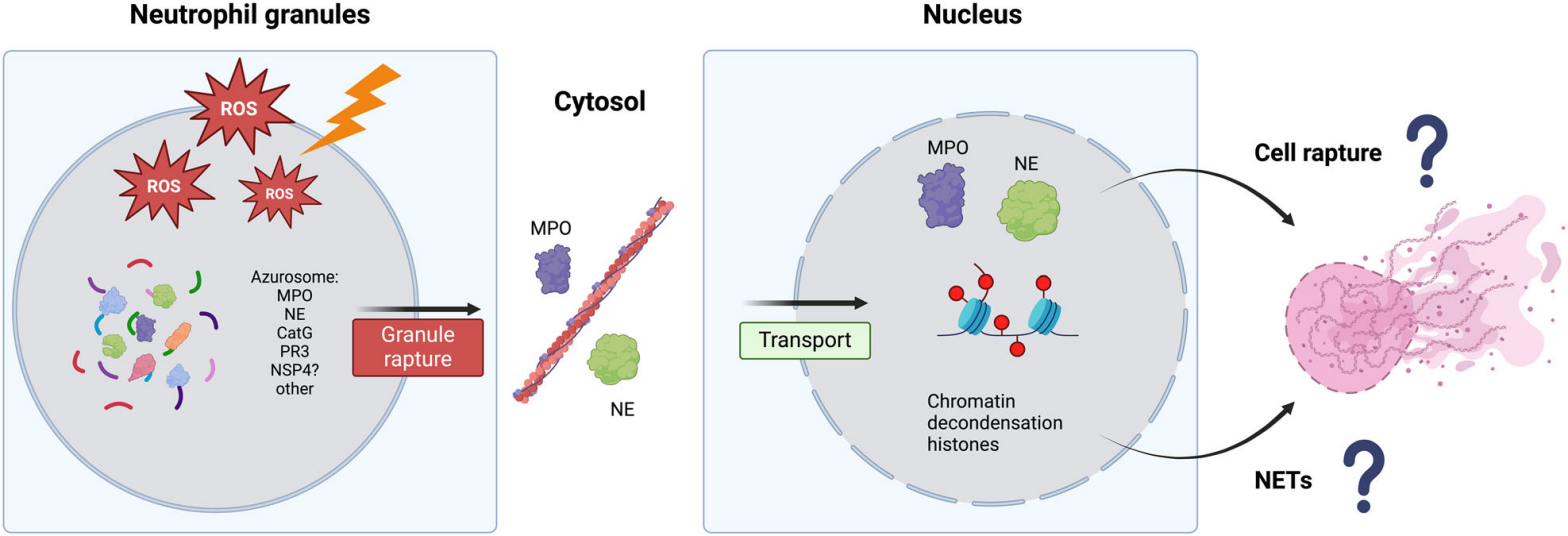
NSPs participate in ROS-induced NET formation. Created with BioRender. In the resting stage of neutrophils, MPO and NE are present in multiprotein complexes called azurosomes, mostly in primary granules. The activation of neutrophils results in the generation of ROS, which leads to pore formation and granule membrane rupture. NE is subsequently released into the cytosol in an MPO-dependent manner. NE binds and processes F-actin filaments, which in turn facilitates NE translocation to the nucleus. In the nucleus, NE cleaves histones, while MPO binds chromatin, which directly contributes to the disintegration of the nuclear envelope and subsequently to cell membrane rupture and NET release.

The generation of ROS by NOX is considered a hallmark of NET formation [20, 32]. Binding of the ligand/pathogen to neutrophil TLR/IgG-Fc receptors causes the release of calcium ions from the ER to the cytoplasm, which then activates PKC, leading to the phosphorylation of gp91phox and enabling NADPH assembly and ROS generation [2, 20, 149]. Upstream signaling pathways, such as the MAPK/ERK, mTOR, PI3K, Akt, and IRAK pathways, have also been implicated in ROS production [134–139]. Accumulating experimental evidence suggests that the ROS-induced MPO-NE axis is a key driver of NET formation [86]. In this context, ROS activates MPO, which subsequently triggers NE translocation from azurophilic granules to the nucleus. NE cleaves histones, while MPO binds to chromatin, leading to chromatin decondensation. In resting neutrophils, MPO and NE are part of the azurosome complex (Fig. 2), which disassembles in response to hydrogen peroxide, releasing NE into the cytosol [87]. Notably, MPO inhibition produces conflicting outcomes, either by delaying NET formation [140] or completely inhibiting NET formation [141]. Once released, NE degrades its own F-actin filaments, facilitating its translocation to the nucleus, where it hydrolyzes histones. PR3 was also shown to promote nuclear decondensation. Inhibition of NE significantly impedes its ability to degrade its cytoskeletal filaments and nuclear translocation[120]. A recent study revealed that another protein, DEK, could act like MPO in the process of chromatin decondensation [150]. The nuclear changes that occur during NETosis lead to disintegration of the nuclear envelope, followed by the intracellular assembly of nuclear and cytoplasmic components. This results in the extrusion of DNA fibers, which are adorned with proteins, into the extracellular space, leading to cell membrane rupture, loss of function, and eventual cell death [17]. Several studies on neutrophils induced by PMA or LPS in mice and humans, respectively, have questioned the necessity of NE for NET formation [141, 143]. However, experiments using NE-deficient models and nonselective NE inhibitors [86, 144–146], along with data from neutrophils in patients with chronic granulomatous disease (CDG), which prevents respiratory burst [17, 140], and Papillon‒Lefèvre syndrome (PLS), which is characterized by mutations in CTSC that prevent NSP activation [147, 148], support the role of NE in NETosis. Interestingly, the recent development of highly selective inhibitors of NE, CatG, PR3, and NSP4 had a minimal effect on NET formation in human neutrophils stimulated with PMA, LPS, *E. coli*, and *S. aureus*, which contrasts with the findings of previous studies [14, 149].

Investigations into the role of NE in NETosis have revealed that NE is not strictly essential for the formation of NETs. Neutrophils isolated from wild-type (WT) and NE-deficient (NE^−/−^) mice exhibited comparable levels of histone H3 hypercitrullination following stimulation with PMA or ionomycin, indicating that PAD4 activity and nuclear translocation are independent of NE function. Although NE^−/−^ neutrophils displayed a moderate (~40%) reduction in NET formation in response to ionomycin, their response to PMA and platelet-activating factor (PAF) was similar to that of WT neutrophils, underscoring the stimulus-dependent role of NE in NETosis.

Interestingly, the elevated NET formation observed in SB1^−/−^ mice was not attenuated by concurrent NE deficiency, suggesting the involvement of additional serine proteases in regulating this process. Furthermore, pharmacological inhibition of NE using sivelestat reduced NET release in both WT and NE^−/−^ neutrophils without affecting histone citrullination, indicating potential off-target effects of the inhibitor, possibly via inhibition of proteinase 3 or other proteolytic enzymes.

From a pathophysiological perspective, the thrombotic phenotype of NE^−/−^ mice diverges markedly from that observed in PAD4^−/−^ mice or in mice treated with recombinant DNase I. In inferior vena cava (IVC) stenosis models, PAD4^−/−^ mice demonstrate significant protection from thrombosis, and the rare thrombi that form are devoid of NET structures. Similarly, DNase treatment, which enzymatically degrades NETs, has been shown to markedly reduce thrombus incidence. These findings suggest that the prothrombotic role of NE may not be primarily attributed to its involvement in NET generation but rather to its activity on already released NETs [151]. Consequently, further studies examining NE’s influence on coagulation pathways, thrombus architecture, and resolution dynamics are warranted to delineate its precise contributions to thrombosis.

However, the importance of other NSPs, including CatG, PR3, and NSP4, has not yet been established. In other forms of cell death that resemble NETosis and are claimed to be NOX/ROS independent, such as “vital” NETosis, NE, or other NSPs may not be required to form NETs.

### Decorating the defense: how NSPs adorn NETs

While there is no consensus about the role of NSPs in NET formation, there is extensive evidence that these enzymes are associated with NETs. NETs are complex structures consisting of DNA (nuclear and/or mitochondrial), histones (H1, H2A, H2B, H3, H4), enzymes (MPO, PAD4; NSPs, including NE, CatG, PR3, and NSP4), peptides, and inhibitors of enzymes or proteases (serpins and cystatins) that are released into the extracellular space upon the activation of neutrophils [2, 3, 7, 10, 20]. Various in-depth analyses of NET composition indicate that dozens of proteins are present within its structure, and the panel of detected proteins varies depending on the stimuli, as summarized in Table 1 [3, 7, 152–154]. Qualitative analysis via nano LCS-MALDI-MS of NETs isolated from PMA-stimulated human neutrophils identified 24 proteins of nuclear, cytoplasmic, and granular origin, including NE, CatG, and PR3. These proteins were further quantified by immunoblotting, and NE was found to be the most abundant nonhistone protein associated with NETs [7]. Additionally, in another mass spectrometry (MS) analysis of PMA-induced NETs, 29 proteins were identified, among which NE and CatG but not PR3 or NSP4 were present [3]. Liquid chromatography–tandem mass spectrometry (LC‒MS/MS) evaluation of NET structures found in the sputum of CF patients revealed 58 proteins, including NE and CatG [152]. Furthermore, other in-depth nano-UHPLC-MS/MS analyses of human neutrophils stimulated with PMA, LPS, and A23187 have been conducted [154]. The proteomic data revealed a total of 330 proteins as NET constituents, 74 of which were present in NETs of all origins, including the spontaneous NETosis control group of neutrophils. Thirteen proteins, namely, CENPE, LYZ, CCT3, HSPA1B, SERPINI2, HRNR, HBA2, NE, RNASE3, LAMP2, PRTN3, SERPINB1, and DNAH5, were found to be common upon induction with PMA, LPS, and A23187, whereas sets of unique proteins for specific stimulation have been recognized and are listed in Table 1. Notably, CatG and Granzyme M (GrM) have also been identified; however, even in the control group, NETosis spontaneously occurred [154]. Recently, a complex study on NET heterogeneity from PMA-stimulated neutrophils derived from healthy human donors over time was conducted. While the total amount of NET-isolated proteins, including NE, CatG, and PR3, was consistent, NSP activity varied from day to day [153]. However, how differences in NET composition impact NET function under healthy, immunological, and diseased conditions remains unclear. To date, NE, CatG, and PR3 have been found to decorate NETs. Since NSP4 is a relatively newly discovered NSP in neutrophils [71], its function and presence in NET structures require intensive investigation. Our group was able to detect NSP4 within NETs; however, NSP4 was in its inactive form [10]. Another study reported the presence of inactive NSP4 in NETs [3]. In addition to the active participation of NSPs in chromatin decondensation, histone modification, and the breakdown of cytoskeletal components to facilitate the release of NETs, the extracellular functions of NETs are believed to be based on their antimicrobial proteolytic activity.

**Table 1:** Diversity in the Composition of NETs.

Neutrophil origin	NETs induction	DNase/MNase treatment	Method	Identified proteins	Reference
Human, healthly	20 mM PMA, 4 h	DNase I	nano LC-MALDI-MS	**NE**, LTF, AZU1, **CatG**, MPO, **PR3**, LYZ, DEFA1, DEFA3, H2AJ, H2BC21,H2BC12L, H3C15, H4C1, MNDA, S100A8, S100A9, S100A12, ACTB, ACTG1, MYH9, ACTN1, ACTN4, LCP1, KRT10, CAT, ENO1, TKT	Urban et al. [7]
Human, healthly	50 mM PMA, 3 h	MNase	LC-MS/MS	ACTB, MPO, LTF, **NE**, HIST2H2BF, H3F3B, HIST1H2AH, AZU1, HIST1H4L, MYH9, TKTL1, ENO1, HIST1H1E, LYZ, **CatG**, S100A9, DEFA1, GAPDH, MNDA-like, GSN, CLC, GPI, PFN1, CORO1A, PGK1, PKM, BASP1, VIM, CFL1, TMSB4X, RNASE3, S100A8, PPIA, GSTP1, TALDO1, Granulins-like, PGD, CAMP, GMFG, HMGB2, LSP1-like, S100A12, Plastin-2-like, ANXA1, TPM2, SH3BGRL3, HMGN2, HP1BP3, MMP9-like, DKFZp686B04128	O’Donoghue et al. [3]
Human, healthly	*P. auerginosa*^a^, 100 min	MNase	LC-MS/MS	TALDO1, TKT, GPI, ALDOA, TPI1, ENO1, PGK1, GAPDH, LDHB, LDHA, PGAM1,MDH1, MDH2, CORO1A, ACTA2, ACTN1, ACTB, PFN1, MYL6B, ARPC1B, GSN, ACTN4, MSN, ACTR3, CAPZA1, HIST1H4A, HIST1H2BA, HIST1H2BB, HIST3H3, HIST1H2BJ, HIST1H2AB, HIST1H2BC, HIST1H3A, HIST1H1A, HIST1H2AD,HIST1H1C, LTF, ERPINB1, MPO, LCN2, LYZ, CAMP, NE, PSMA1, ANXA1, ANXA3, PPIA, HSPA8, HSPA1A, HSPA1L, MMP9, HSPE1, HSPA5, ANXA5, CAT, PRDX2, PDIA3, PRDX1, SOD1, UBA52, S100A12, RETN, FTL, YWHAG, FTH1, PDIA3, YWHAE, SET, ANP32A, NAA38, S100A4, ARHGDIB	Dwyer et al. [152]
Human, CF sputum	*P. auerginosa*	DNase I	LC-MS/MS	GAPDH, TPI1, CORO1A, MYH9, GSN, PFN1, ACTN1, FLNA, ACTN4, LTF, MPO, EPX, **CatG**, PGLYRP1, AZU1, CAMP, **NE**, LYZ, S100A12, S100A4, BPIFB2, ANXA1, ANXA3, ANXA4, HSPA8, ANXA6, HSPA1A, HSPA1L, HSPA2, HSPA5, CAT, **PR3**, PLBD1, CHI3L1, MMP8, MMP9, SERPINA3, PPIB, QSOX1, SERPINB10, SERPING1, SERPINA1, TKT, TALDO1, VCL, ITGAM, AZGP1, C3, LRG1, CRISP3, HPX, IQGAP1, LCP1, A1BG, MUC5B, LCN2, CAP1	
Human, healthly	100 mM PMA, 3 h	DNase I	nano-UHPLC-MS/MS	ACTR3C, STX1A, CS, OGDH, SYNJ1, NXN, HSPA12A, ASAH1, KRT10, ADD2, NAP1L4, NCOA1, GRIA2, CALB1, RAB1B, SKP1, GPD2, SLC8A2, PACSIN1, DLST, EIF3G, CLIC1, TPPP, CA2, GNAI1, ATP1A1, SLC25A6, AKR1A1, CDH2, COX5A, UBA1, HSPA9, RBM11, AKAP6, SHB, SETX, PKD1L2, HIST2H2AB ^b^	Petretto et al. [154]
	1 µg/ml LPS, 3 h			SIRPA, EPX, ORM1, HAUS5, HUS1, PRPF40B, CCDC154 ^b^	
	4 µM A23187, 3 h			ATAD5, GPI, IQGAP1, ELP3, MYL6, WDR1, CHIT1, NLN, ANXA5, CEP112, UACA, COPG1, UNC13D, MAN2B1, AKAP8, CSTB, UBTFL1, SPTB, HSP90B2P, CAPZA1, DST, CLLU1OS, TMEM69, BSPRY, RAD50, ZNF251, AKAP8 ^b^	
Human, healthly	25 mM PMA, 4 h	No	FRET enzyamtic activity (NE, CG, PR3), MPO activity, flow cytometry (cytokine panel)	**NE,** **CatG,** **PR3**, MPO, IL-8, IL-1RA, G-CSF, IL-1α, IL-1β, TNF-α	Collins et al. [153]

^a^ Various strains.

^b^ Only unique proteins, not found in other treatment groups or the spontaneously NETotic group.

### Breaking down the barriers: substrates in NSP-driven NETosis

To elucidate the intricacies of NET formation, it is essential to consider the involvement of a myriad of proteins and enzymes. This chapter meticulously examines the identified substrates of NSPs, including PAD4, gasdermins (GSDMs), and histones, involved in this phenomenon.

Histones, distinguished by their high basicity and abundance of Lys and Arg residues, are ubiquitous constituents of eukaryotic cell nuclei [155]. DNA wraps around histones to form nucleosomes, which further condense into densely packed chromatin structures. The five families of histones, categorized into linker histones (e.g., histone H1) and core histones (e.g., histones H2a, H2b, H3, and H4), are susceptible to enzymatic hydrolysis, particularly by NSPs and especially NE [156–158]. This enzymatic action facilitates the unwinding of DNA within the nucleus or mitochondria, rendering histones pivotal substrates for NET formation. Moreover, histone H1 is a substrate for the serine protease GrA, which could play a role in NETosis [159]. GrA is a key player in caspase-independent cell death; therefore, it may be involved in different types of cell death, such as apoptosis. The intricate process of histone cleavage involves chromatin decondensation, which is characterized by decreased heterogeneity and increased DNA occupancy within the cellular space during NETosis [11]. Central to this process is PAD4, a calcium-dependent enzyme primarily responsible for catalyzing citrullination, a posttranslational modification that reduces the affinity between histones and DNA [160, 161]. This modulation prompts histone dissociation from DNA, leading to the loss of chromatin compaction. Notably, histone posttranslational modifications, including citrullination [121] and acetylation, are pivotal for effective NETosis. Among these modifications, histone citrullination by PAD4 has emerged as a well-characterized event crucial for DNA stabilization [162].

The family of peptidyl arginine deiminases (PADs) contains five enzymes in humans and mice, and PAD4 prominently expressed in granulocytes, cancerous cell lines, and tumors, further underscoring the importance of calcium in PAD-mediated citrullination. Studies have revealed a correlation between elevated calcium levels and PAD enzymatic activity, suggesting that calcium flux is a requisite for optimal PAD function. Moreover, PAD4 deficiency in mice was shown to be correlated with impaired citrullination of histones and subsequent inhibition of NETosis triggered by various stimuli. Pharmacological inhibition of PAD4 verified its essential role in histone citrullination and NET release [163].

Emerging evidence suggests that, in addition to their antimicrobial functions, neutrophils contribute to the amplification of host inflammatory responses. Activated neutrophils can release cytokines and chemokines, attracting peripheral blood monocytes/macrophages to sites of inflammation [164]. Furthermore, the release of serine proteases from neutrophil granules increases the levels of processed interleukins (ILs), particularly those from the IL-1 cytokine family, released from damaged tissues. Studies have shown that neutrophil-associated proteases—namely, NE, PR3, and CatG—process and activate interleukins such as IL-36α, IL-36β, IL-36γ, and IL-1α [9–11]. Among these, CatG has the most potent activity.

Understanding the interaction between NET formation and interleukin release is critical for understanding inflammatory and autoimmune diseases such as rheumatoid arthritis, systemic lupus erythematosus (SLE), and cystic fibrosis. The role of the cytokine IL-36 in inflammatory disorders, particularly psoriasis, is noteworthy because of the high neutrophil infiltration in the affected tissues [12]. Psoriasis, a chronic skin disease, is characterized by plaques rich in neutrophils, macrophages, dendritic cells, and T cells, creating a proinflammatory environment [13]. Martin’s group identified specific protease cleavage sites in IL-36β and IL-36γ targeted by CatG and NE, with mutations at these sites preventing interleukin activation. Experiments using NET-stimulated neutrophils confirmed that CatG was the primary activator of IL-36β, whereas NE played a minimal role. Inhibiting CatG suppressed IL-36α and IL-36β activation, whereas NE inhibition had little effect. Surprisingly, the inhibition of both CatG and NE reduced IL-36γ activation, challenging the assumption that NE is the primary activator of IL-36γ [9].

The involvement of GSDMs in extracellular NET release is a topic of interest. GSDMs, known as pore-forming proteins that are implicated in inflammation, septic shock, and autoimmune diseases, induce the formation of pores (~21 nm diameter) in the plasma membrane, resulting in osmotic imbalance, cell lysis, and proinflammatory cytokine release [165, 166]. While traditionally associated with pyroptosis, gasdermin-D (GSDMD) cleavage by caspases prompts pore formation. However, recent investigations have suggested a potential role for GSDMD in NET formation, potentially facilitated by its ability to induce pore formation before the cytoplasmic membrane is compromised [35], which contrasts with the findings of the latest studies by Simon’s group, which presented data indicating that NET formation is independent of GSDMD cleavage and pyroptosis [167]. However, a previous study reported serine protease-mediated cleavage of certain GSDM types, including GSDMD, GSDME, and GSDMB, in neutrophils [168]. A study using the GSDMD inhibitor LDC7559 highlighted the downstream effects of inhibiting GSDMs on NET formation, with evidence suggesting direct binding of LDC7559 to GSDMD [169, 170]. The efficacy of this inhibitor in reducing NET formation underscores the critical role of GSDMD in this process. Additionally, several studies have indicated a possible role for GSDMD as a substrate for NE, further elucidating its involvement in NETosis [35, 171].

In summary, understanding the roles of various NSP substrates in NET formation provides crucial insights into the molecular mechanisms underlying this immune process. It is plausible that pore formation by GSDMs may influence NET release by providing a route for DNA to escape the cell. These insights are essential for developing therapeutic interventions targeting dysregulated NETosis, highlighting the need for a thorough understanding of the complex interactions between proteins and enzymes involved.

### Active defense: the role of NSPs within NETs

At the beginning of NET research, it was assumed that the NSPs decorating the extruded DNA determined NET proteolytic activity. It is commonly believed that extracellular, released, and trapped (by extruded DNA) NSPs exhibit enzymatic activity to hydrolyze microbial components such as bacterial cell walls, outer membrane proteins, and virulence factors. Indeed, several data support the notion that the proteolytic activity of NSPs aids in neutralizing pathogens trapped by NETs and limiting their spread within the host [2, 7, 20]. Subsequent studies have used highly selective fluorescent substrates of NE, CatG, and PR3 to determine proteolytic action in CF patients as well as neutrophils stimulated with *S. aureus* and *P. aeruginosa* and revealed that DNase and nuclease treatment increases the activity of NSPs [131]. In 2013, O’Donoghue and coworkers performed substrate profiling of proteases via an MPS-MS assay to determine that NE and, to a lesser extent, CatG and PR3 are active in NETs derived from PMA-stimulated neutrophils. Additionally, NE immunization results in increased proteolytic activity of CatG as a mechanism of compensation [3]. In a recent study of PMA-induced human neutrophils, FRET fluorogenic NSP substrates were used to reveal variations in the activity of NE, CatG, and PR3 over time [153]. Additionally, oxidative processing increases the activity of MPO, ADAMTS13, and collagenase [172–174], which may suggest that ROS could increase the activity of NSPs in a similar manner. In addition, it has been reported that the release of extracellular histones and DNA not only restricts microbial growth but is also able to degrade microbes [2, 175], which raises the question of whether the proteolytic activity of NSPs is truly involved in NET function. Moreover, our latest data support the idea that NSPs present in NET structures are not active at all. The sensitive and specific technology of activity-based protein profiling enables the distinction between active and nonactive enzymes [84, 176]. First, using designed biotinylated probes against NE or NSP4 and freshly isolated human neutrophils or recombinant protein, we demonstrated that those NSPs indeed decorate NETs; however, they are not active or exhibit very low activity levels (below the detection limit), whereas they are still active when they are present in azurophilic granules [84, 177]. Additionally, labeling with specific probes has revealed that the active forms of those NSPs remain intracellular, whereas the minimal concentration of proteases found to decorate NETs are inactive [10, 178]. This may be because DNA-binding NSPs possess a high pI (isoelectric point) and block access to their active site cleft. Moreover, another group has developed FRET-based reporters consisting of a DNA binder to target the activity of NE and CatG. Experiments with PMA-induced NETs revealed that NE localized to the nucleus has proteolytic activity [179]. These inconclusive results indicate the complexity of the NSP activation-inactivation mechanism and require further research. Additionally, these findings prompt reconsideration of the existing paradigm regarding NSP activity in NETotic cell death.

## Concluding remarks and future perspectives

NET formation is a complex phenomenon that is still controversial, particularly regarding its overlapping aspects with other forms of cell death, the role of NSPs, the nature of scaffolding DNA, and the possible involvement of other factors.

Undeniably, research on NET formation presents many challenges, starting from the short lifespan of neutrophils, their ability to be handled and their ability to be activated through a broad spectrum of NETosis activators (both un-physiological and physiological) causing different effects and the activation of multiple pathways, and methodological limitations and sophisticated data analysis requirements. Presumably, this complexity reflects the physiologically determined need for protection against various pathogenic factors. The multiple compounds present in NET structures, which are often conditioned by various stimuli, make it difficult to identify which component contributes to a specific effect. Additionally, capturing the dynamics of the process, which involves the translocation and release of diverse proteins, requires a variety of highly specific reagents and high-resolution equipment. Additionally, we cannot exclude the possibility that the substrates or inhibitors of these NET components may have been degraded during the time course of the experiments. Addressing these limitations often requires a multidisciplinary approach combining biochemical assays, modern imaging techniques, and both animal and clinical studies.

Furthermore, the features of various types of cell death are similar, such as disturbances in the plasma membrane, nuclear and cytoplasmic changes, and the involvement of key factors. Additionally, the identification of new substrates/interacting proteins that play a role in several different cell death pathways makes analysis even more difficult. The complexity of both mechanisms and experimental setups may lead us to unintentional but incorrect interpretations. Therefore, it is extremely important to look critically at the design and evaluation of conducted studies.

Despite many years of investigations on cell death, the fundamental question in the field is still when and how we define a cell as dead. According to the guidelines of the Nomenclature Committee on Cell Death, a cell can be defined as dead when (i) it has lost its plasma membrane integrity and/or (ii) it has undergone complete disintegration, including its nucleus, and/or (iii) its compounds/fragments have been engulfed by other cells [33]. In addition, the proposed definition of NETosis is strongly related to ROS generation and NET extrusion, which lead to cellular death [180]. Naming every form of DNA extrusion that results in cell ETosis (since this phenomenon has been observed in various types of cells, not just neutrophils) would be unwise. In particular, “vital” NETosis is controversial because (i) it fails to fulfill the requirement of ROS dependency in NET formation, (ii) it does not meet the criteria of dead cells, and (iii) the cells remain alive/vital. It is difficult to consider a cell deprived of some DNA and functional proteins as capable of performing basic functions. In addition, what would be the benefit of a retaining a cell that could not perform its basic processes? Observations of crawling neutrophils in that case suggested only a short-term possibility of migration and chemotaxis [22, 101]. This phenomenon of rapid neutrophil action could be considered an evolutionarily old mechanism of the defense response to harsh stimuli. In the case of classical NETosis, the duration of response is longer, which can allow pathogens to spread the infection. In addition, this prolonged response time allows the pathogen to develop defense mechanisms against NETs, such as encapsulation to decrease recognition by NETs, the production and secretion of endonucleases to destroy DNA, and the expression of specific proteins that inhibit NSP activity [181–184]. We believe that a more appropriate approach would be to consider the processes of suicidal and “vital” NETosis; however, these processes share few similarities.

**Fig. 3:**
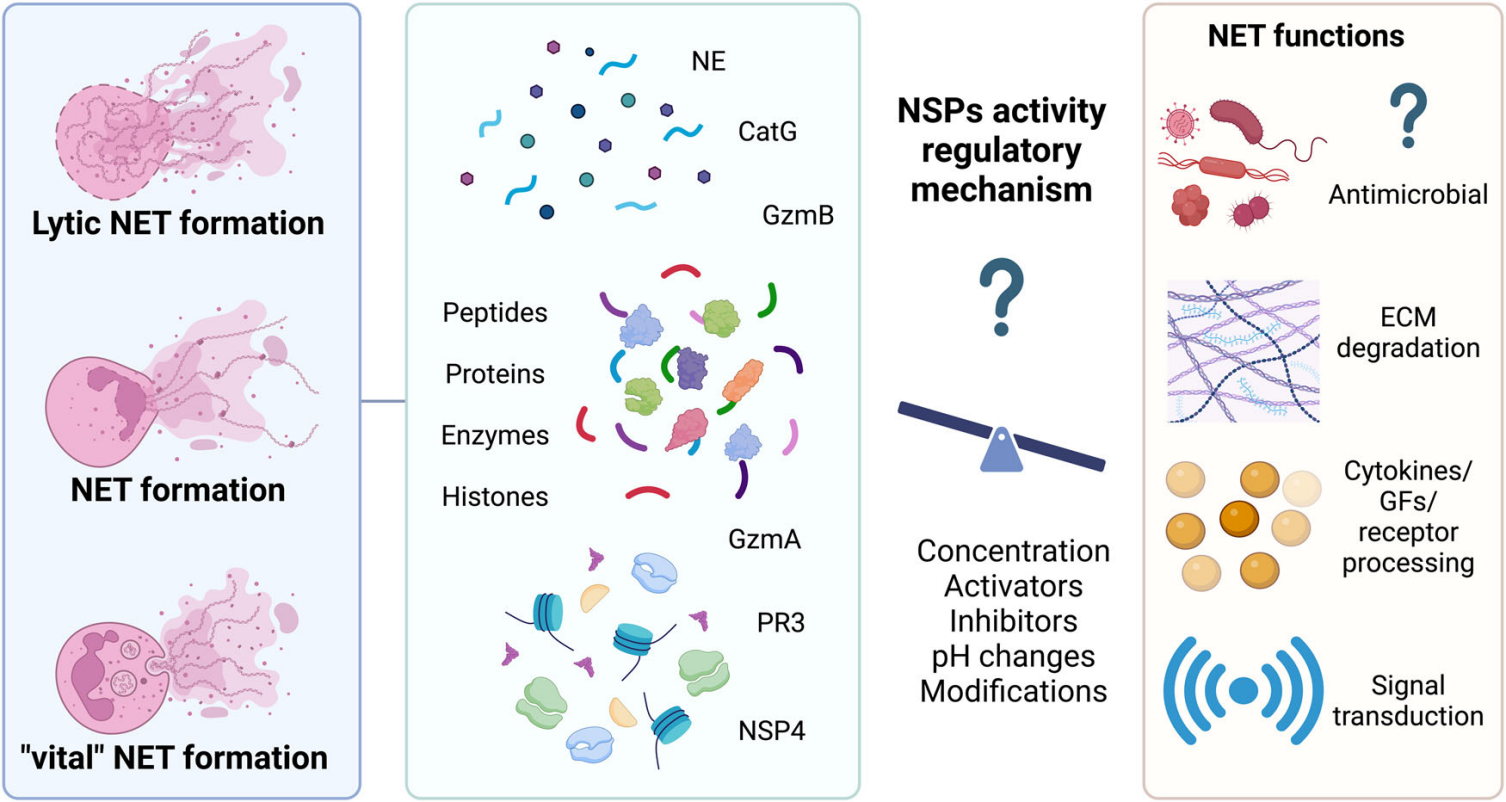
Schematic representation of the regulation of NSP activity and their suggested functions. Created with BioRender. The form of cell death associated with the extrusion of DNA together with the formation of granular and cytosolic proteins, is named NETosis. In addition to DNA, NETs consist of a great variety of peptides, histones, proteins, and enzymes. Among them, NSPs are proposed to play a role in the antimicrobial features of NETs through their proteolytic activity; extracellularly, these enzymes can also degrade ECM components, process cytokines, GFs, and their receptors; and act as signaling molecules. The mechanisms controlling the activity of NSPs might include the interaction of NSPs with their inhibitors and/or activators, autoinhibition caused by too high a concentration of NSPs, environmental pH changes affecting the catalytic centers of enzymes, and their posttranslational modifications.

Despite the lack of strong consensus that NETs contain enzymatically active NSPs, we believe that, in light of recent reports, the functionality of NSPs in NETs should be reconsidered [10, 84, 177]. The statement that proteases lose activity after being released from inside the cell while trapped in NETs is based on studies using highly specific probe substrates that allow monitoring of protease activity over time. Conflicting results concerning NSP activity suggest the need for in-depth analyses, considering not only highly specific and verified substrates but also accurate confirmation of their localization and correct control, eliminating the risk of artificial activity [131, 153, 179]. In particular, when sample preparation includes DNase or MNase treatment, it has been reported to activate or increase the enzymatic potential of NSPs [3, 131]. We do not question the validity of using DNase to separate and isolate proteins from the DNA within NETs, but it must be considered that such treatment may affect the measurement of enzyme activity. The use of advanced live imaging systems together with novel technologies, such as FRET-based reporters, ABPs, and specific inhibitors, could be extremely useful [6, 178, 179, 185, 186]. Further studies are needed to elucidate the mechanism responsible for the inactivation of NSPs. The factors regulating this process are as follows: (i) interactions with other extracellular proteins, including inhibitors of NSPs; (ii) excessively high extracellular concentrations of NSPs resulting in autoinhibition; (iii) changes in the environmental pH affecting the catalytic centers of NSPs; or (iv) posttranslational modifications of NSPs and their substrates, as suggested in Fig. 3. Additionally, we cannot exclude that NSPs are simply trapped by DNA networks via electrostatic interactions as a purely biophysical effect of NSPs being captured and attached to DNA from the intracellular mixture of molecules during the release of NETs [187]. Another challenging aspect of NSP inactivation is whether this process is reversible and, if so, how. Almost two decades ago, it was demonstrated that DNA bound to NE and CatG causes a decrease in their activity, probably due to ionic interactions [187, 188]. An existing report that NE, a compound of NETs after DNase treatment, was able to process substrates suggested that, after NET degradation, NSPs could be reactivated [131]. Thus, we cannot exclude the existence of extracellular NSP-activating and -inactivating mechanisms. Furthermore, one may wonder whether NSPs released from NETs can be transported to locations where they are needed and become active again. The activity of the trapped and inactive NSPs can be recovered after MNase treatment. It may also be that only some NSPs are caught in traps, and some are secreted independently from NETs, with or without granules, and are not caught in traps. Ultimately, the function of inactive NSPs in NETs should be considered. It is highly probable that this is a primitive defense mechanism to avoid damage to surrounding tissues and prevent uncontrolled inflammation. We cannot exclude the possibility that DNA in NET structures captures released proteases to protect the surrounding cells from proteolysis. Additionally, reducing the role of NSPs only to their proteolytic activity would not be beneficial. In addition to their ability to degrade ECM components and/or process cytokines, growth factors and receptors [50, 140–146], they can also perform other functions unrelated to lysis, such as acting as signaling molecules, as proposed in Fig. 3.

A growing body of evidence implicates NSPs and NET formation in many pathological states and diseases, including diabetes, arthritis, thrombosis, sepsis, COVID-19, neutropenia, cystic fibrosis, Alzheimer’s disease, and several types of cancer, such as acute promyelocytic leukemia and colorectal or breast cancer [97, 101, 106, 107, 112–117]. The levels of NETs and NSPs are increasingly used as biomarkers of certain pathological states [105, 118, 189]. The negative roles of NSPs may include (i) exerting cytotoxic effects in sepsis and injury, (ii) promoting the autoimmune response, (iii) facilitating tumor metastasis and progression, and (iv) promoting thrombosis formation. Owing to the aging of society and the increase in disease rates, understanding the mechanisms that activate and inactivate NSPs in these pathological conditions is extremely important. A better understanding of the mode of action of NSPs, especially those involved in NET formation, might lead to the development of new diagnostic and therapeutic strategies. The development of tools for the specific and precise measurement of enzyme activity, especially NSPs, will provide many opportunities to explore the intricate mysteries of NETotic cell death.
